# Expanding the clinical utility of a pathomics-based AI model for predicting 9p loss in oral leukoplakia and head and neck squamous cell carcinoma

**DOI:** 10.1097/JS9.0000000000002956

**Published:** 2025-07-08

**Authors:** Jing Li, Jian Chen, Zhongjun Yang

**Affiliations:** a Department of Stomatology, Qilu Hospital (Qingdao), Cheeloo College of Medicine, Shandong University, Qingdao, China

*Dear Editor,*

We comment with great interest, the recent article titled “Identification of genomic alteration and prognosis using pathomics-based artificial intelligence in oral leukoplakia and head and neck squamous cell carcinoma: a multicenter experimental study^[1]^,” published in the *International Journal of Surgery*, which reported the development of an artificial intelligence (AI) model (9PLP) to predict chromosome 9p loss based on routine hematoxylin and eosin (H&E)-stained images. This model was further extended to assess prognosis in patients with head and neck squamous cell carcinoma (HNSCC). The authors applied a sophisticated approach combining the transformer neural network and XGBoost algorithms, achieving high efficiency in genomic alteration prediction while minimizing cost and technical barriers. This approach holds particular promise for resource-limited clinical settings and offers a novel strategy for the individualized management of oral potentially malignant disorders (OPMDs) and HNSCC, and all contents comply with the TITAN 2025 guidelines^[2]^.

As oral and maxillofacial surgeons with extensive experience in the comprehensive management of oral leukoplakia (OLK) and oral squamous cell carcinoma (OSCC), we recognize the innovation and potential clinical value of this study. However, based on our clinical observations, we would like to offer several supplementary perspectives.

First, it is widely accepted that the malignant transformation of OLK is driven by multiple genomic events rather than chromosome 9p loss alone. Alterations such as 3q gain, TP53 mutations, NOTCH1 inactivation, and CDKN2A deletion also play important roles at various stages of disease progression^[3]^. We suggest that incorporating these molecular features into future iterations of the AI model could enhance its predictive accuracy and reduce the risk of over-reliance on a single biomarker.

Second, the tumor immune microenvironment has become increasingly important in prognosis and therapeutic decision-making for HNSCC. Biomarkers such as PD-L1 expression, tumor-infiltrating lymphocyte (TIL) density, and co-deletion of JAK2 and PD-L1 have all been linked to immune response and treatment outcomes^[4]^. The current 9PLP model does not include immune-related features. We encourage the integration of immunohistochemistry, transcriptomic data, or spatial multi-omics to expand the model’s clinical applicability, particularly in the context of precision immunotherapy.

Third, we observed that the majority of OLK samples used to train and validate the 9PLP model were from a single northern Chinese center (310 out of 333 cases), with only 23 external samples from southern China. While the model demonstrated promising generalizability, this geographic imbalance may limit its applicability across more diverse populations. Significant regional differences exist in lifestyle habits (e.g., tobacco and alcohol use, areca nut or lime chewing), HPV infection patterns, and oral hygiene status, which could affect both molecular pathogenesis and histological appearance^[5]^. Moreover, variation in tissue processing, H&E staining protocols, and digital slide scanning across institutions may further impact model robustness. We advocate for future studies to include multicenter datasets from different geographic regions, ideally under prospective design, to improve the model’s reproducibility and clinical adaptability.

Finally, the underlying mechanism by which the AI model links H&E image features to chromosome 9p loss remains unclear. While the model exhibits high predictive performance, it is uncertain whether it detects direct histological consequences of 9p deletion, or rather co-occurring morphological traits such as epithelial dysplasia, inflammatory infiltration, or nuclear atypia. There is a risk that the model “remembers” these surrogate features instead of the actual molecular event. Future studies employing spatial transcriptomics or multimodal imaging could help elucidate the causal relationship between the visual features extracted by the model and the underlying genetic alterations, thus enhancing the biological interpretability of the AI predictions.

In summary, *Cai et al*. have proposed a novel and highly promising model for AI-assisted molecular prediction in OPMDs and HNSCC. We encourage further expansion of the training dataset to include more diverse regional and molecular profiles, as well as mechanistic validation to ensure clinical reliability. Such efforts will be critical for translating the 9PLP model from research into routine clinical practice, ultimately supporting precision diagnosis and management in oral oncology.

## Data Availability

Not applicable.
